# Excision of the Rectum for Carcinoma

**Published:** 1877-04

**Authors:** R. J. Levis

**Affiliations:** Penn Hospital, Philadelphia


Excision of the Rectum for Carcinoma.—*Dr. R. J. Levis, Penn Hospital, Philadelphia.*—The patient, upon whom this formidable operatiou was performed, was a man sixty years of age, who had had symptoms of rectal trouble for about one year. Digital examination showed that there was a nodulated mass occupying the anterior part of the rectal wall, and extending a short distance to the sides. The finger could easily be hooked over the top of the tumor, which, therefore, extended about two and a-half inches up the bowel. After the bowels had been evacuated by a laxative and an enema, the operation was done as follows: After a large bougie had been introduced into the bladder to serve as a guide to the position of, and also to steady the urethra, an incision was made from the base of the scrotum to the coccyx, encircling both sides of the anal aperture. The hand of the operator was then introduced behind the rectum, into the hollow of the sacrum, by which means the bowel was torne loos from its posterior attachments. After this was done, Dr. Levis, by means of his finger and the serrated scissors, broke down the attachment of the viscus around to the front. The cancerous portion was then carefully dissected from the prostate gland and lower part of the bladder, exposing these structures perfectly to view. During these procedures, the vessels were promptly ligated as soon as divided, and double sutures passed through the skin into the rectum above the proposed line of excision. These were not fastened, but left in position in order to give perfect control of the parts.

When the diseased portion had been thoroughly isolated, the gut was drawn forcibly downward by seizing the tumor, and the scissors used to cut through the walls of the bowel. About two and a half inches were thus excised, leaving behind a perfectly soft and smooth mucous surface. The sutures were then shotted, and a few extra ones applied to keep the gut in position, which was, by this means, securely stitched to the surrounding integument. The parts were washed and dressed with carbolized solutions, and, at present, the patient is doing exceedingly well.

This case reminds me of a case of colotomy done in this hospital a few weeks ago, by Dr. T. G. Morton. The woman, when admitted, had such a tight cancerous stricture, that defecation had been impossible for some time. Colotomy was determined upon, and the colon was opened in the left lumbar region. A most curious complication was the finding of a large cyst, probably renal, lying exactly where the colon was expected, and producing a good deal of displacement of parts, which rendered the operation a very difficult one. The patient, who was a very poor subject, died about two days after the operation.—*Archives of Clinical Surgery.*

